# Addressing the research gap: access to care hinders genetic discovery in systemic lupus erythematosus patients throughout the African diaspora

**DOI:** 10.3389/fgene.2024.1414490

**Published:** 2024-08-15

**Authors:** Jihwan Hwang, Ida Dzifa Dey, Olusola Ayanlowo, Cindy Flower, Amanda King, Nicole Johnson, Uyiekpen Ima-Edomwonyi, Hakeem Olasebikan, Titilola Falasinnu, Vishnuprabu Durairaj Pandian, Ashira Blazer

**Affiliations:** ^1^ Icahn School of Medicine at Mount Sinai, New York, NY, United States; ^2^ Department of Medicine, Division of Rheumatology, University of Ghana, Accra, Ghana; ^3^ Department of Dermatology, College of Medicine University of Lagos, Lagos, Nigeria; ^4^ Department of Medicine, Division of Rheumatology, The University of the West Indies, Cave Hill, Saint Michael, Barbados; ^5^ Division of Rheumatology, Bay Medical Centre, Castries, Saint Lucia; ^6^ Department of Pediatrics, Division of Rheumatology, University of Calgary, Calgary, AB, Canada; ^7^ Department of Medicine, Division of Rheumatology, College of Medicine University of Lagos, Lagos, Nigeria; ^8^ Department of Anesthesiology, Perioperative and Pain Medicine, Stanford School of Medicine, Stanford, CA, United States; ^9^ Department of Medicine, Division of Rheumatology, University of Maryland School of Medicine, Baltimore, MD, United States

**Keywords:** systemic lupus erythematosus, health disparities, global health, African diaspora, lupus introduction ancestry and race

## Abstract

Systemic lupus erythematosus (SLE) is a complex autoimmune condition that disproportionately impacts non-White ethnic and racial groups, particularly individuals in the African diaspora who experience heightened incidence, prevalence, and adverse outcomes. Genetic and epigenetic factors play significant roles in SLE risk, however these factors neither explain the whole of SLE risk nor the stark racial disparities we observe. Moreover, our understanding of genetic risk factors within African ancestry populations is limited due to social and environmental influences on research participation, disease presentation, and healthcare access. Globally, the African diaspora faces barriers in accessing essential SLE diagnostic tools, therapeutics, healthcare practitioners, and high-quality clinical and translational research studies. Here, we provide insights into the current state of genetic studies within African ancestry populations and highlight the unique challenges encountered in SLE care and research across countries of varying income levels. We also identify opportunities to address these disparities and promote scientific equity for individuals affected by SLE within the global African diaspora.

## Introduction

### Ancestry and race

In this manuscript, we discuss genetic ancestry, which refers to the paths through which individuals inherit DNA from specific ancestors across the generations; and genetic similarity, which is the measure of genetic relatedness between individuals or groups of individuals (Mathieson and Scally, 2020; Lewis et al., 2023). We will also discuss ethnicity, identity, and race which are intricate labels shaped by visible traits like skin color, as well as cultural, economic, geographical, and social influences (Khan et al., 2022). The biological and social sciences have reached a clear consensus that race is a social construct rather than a biological attribute (Mathieson and Scally, 2020; Lewis et al., 2023). While racial categories may loosely correlate with genetic similarity, race as a measure of biological diversity in humans is a misconception. Racial and ethnic categories encompass physical traits, but also culture, language, religion, and identity (Khan et al., 2022). Human adaptation to selective pressures has influenced genomic diversity, impacting genetic heterogeneity both within and between races (Khan et al., 2022). Importantly, the stark health differences we see in SLE outcomes throughout the African diaspora are due to differences in the ability of these racialized groups to seek and maintain adequate healthcare (Parodis et al., 2023). When considering genetic influences on SLE outcomes, we must therefore adequately appreciate the unique social determinants of health impacting this world population (Parodis et al., 2023). Here we will discuss both updates in genetic epidemiology across the African diaspora, and the many health barriers that cause regional racial and ethnic disparities.

### Regional epidemiology

Systemic lupus erythematosus (SLE) disproportionately affects women and non-White racial and ethnic groups--particularly women in the African diaspora during childbearing years (Barber et al., 2023). However, the epidemiology of SLE across the African diaspora is poorly understood due to access to care challenges, case ascertainment bias, and inconsistent epidemiologic methodologies (Barber et al., 2023). The incidence and prevalence of SLE varies across North America by region, sex, and racial groups. In the CDC’s National Lupus Registry network, the overall estimated prevalence of SLE at 72.8 per 100,000 person-years in the United States (Izmirly et al., 2021). Stark racial and ethnic disparities across regions were shown with prevalence rates among African American women reaching 230.9 per 100,000 person-years (Izmirly et al., 2021). In the Georgia Lupus Registry Report, incident and prevalent SLE cases among African Americans exhibited earlier mortality at 51.8 and 52.8 years respectively compared to White SLE cases (64.4 and 65.0 years) (Lim et al., 2019). Similarly in a multiethnic, longitudinal, observational cohort study in The University of Toronto Lupus clinic showed, Afro-Canadians had a higher prevalence of renal disease and damage at an Odds Ratio of 1.64 compared to White Canadians (1.0) and Canadian Chinese (1.02) (Johnson et al., 2006). In a large multiethnic cohort: the 1,000 Canadian faces of Lupus showed the frequency of SLE diagnosis varied across four ethnic groups with 59% and 56% of Asian and Afro-Caribbean participants exhibiting renal involvement compared to 40% of White Canadians (Peschken et al., 2009).

Limited epidemiologic studies on SLE in the Caribbean have been published, but data from higher-resourced territories offer valuable insights. For instance, in Barbados, the first total population study of SLE in the English-speaking Caribbean between 2000 and 2009 demonstrated an incidence of 12.21 per 100,000 person years in women and 0.84 per 100,000 person years in men (Flower et al., 2017). Studies in St. Lucia and Martinique have estimated an SLE incidence of 8.0 per 100,000 person years and 4.7 per 100,000 person years respectively (King, 2016). Furthermore, survival rates vary considerably across territories due to differences in health infrastructure and access to specialized care. For example, Barbados reported an overall 5-year survival rate of 79.9% with 91% survival among patients without nephritis, and 68% in patients with nephritis (Flower et al., 2012). Martinique, a French territory with specialized SLE centers and socialized healthcare, exhibited higher survival rates over a 10, 15, and 20-year period at 95.5%, 94.4%, and 92.1% respectively (Flower et al., 2012). St. Lucia, with a private rheumatology practice emphasizing patient education and empowerment, reported impressive 5-year survival rates at 97%, particularly among non-impoverished participants (King A et al., 2023).

In Africa, the prevalence of SLE remains poorly established, primarily due to the absence of accurate estimates from longitudinal studies. Previous notions of a “lupus gradient,” suggesting lower prevalence rates in Sub-Saharan Africa compared to Western countries, have been challenged by recent regional publications (Adelowo and Oguntona, 2009; Adelowo et al., 2021; Emorinken et al., 2021; Fatoye et al., 2022; Osaze et al., 2023). Hospital-based studies and initiatives like the Lupus Registry in Nigeria shed light on the epidemiology of SLE, revealing a departure from previous passive ascertainment methods. A recent systematic review and meta-analysis of 15 pooled hospital-based studies found that SLE represented 1.7% (95% CI, 0.8%–2.9%) of 28,375 total hospital admissions (Osaze et al., 2023). The female-to-male ratio has been estimated between 8.7:1 and 32.3:1 with the average age of presentation between 29.2 and 36.6 years (Adelowo and Oguntona, 2009; Osaze et al., 2023). Moreover, the recently established Lupus Registry in Nigeria (LURIN), funded by ILAR (the International League of Associations for Rheumatology), marks a significant departure from previous passive ascertainment methods (Odunlami et al., 2024). Passive capture, which identified only 52 cases over a 5-year period, were limited in capturing the full scope of SLE cases (Osaze et al., 2023). In contrast, LURIN actively identifies cases and has already recorded 746 SLE cases within just 12 months (Emorinken et al., 2021). This remarkable increase underscores the critical importance of dedicated funding for SLE epidemiology initiatives in low- and middle-income countries.

Difference in SLE incidence between women and men may reflect both SLE pathogenesis and case missingness due to cognitive biases among physicians (Simard et al., 2022). The literature reports that 90% of SLE patients are female (Weckerle and Niewold, 2011), therefore, physicians may have lower clinical suspicion for SLE in presenting male patients (Simard et al., 2022). The SLE incidence rate has been reported at 0.4 for White males, 3.5 for White females, 0.7 for African American males, and 9.2 for African American females (McCarty et al., 1995). The larger difference in SLE incidence between males and females across these racial groups may suggest that the detrimental effect of SLE underdiagnosis among males affects African American patients more than White patients (Simard et al., 2022). Misdiagnosis or delayed diagnosis among men may lead to gender disparities in disease severity and quality of life. For instance, male patients with milder symptoms may not be diagnosed only later to present with end-organ manifestations of SLE; ultimately perpetuating the narrative that male SLE patients have more severe clinical presentations (Simard et al., 2022). However, numerous factors can contribute to the observed disparities in SLE incidence by sex, and the most influential factors that lead to poorer outcomes among male SLE patients remain unclear.

### Genetics

Several lines of evidence support a genetic susceptibility toward SLE, including a higher reported concordance between monozygotic (24%–56%) than dizygotic (2%–4%) twins (Reid et al., 2020). While inheriting one or more risk alleles may explain an individual’s disease susceptibility (Reid et al., 2020), genetic ancestry cannot explain SLE susceptibility between racial or ethnic groups. The over 130 SLE associated risk alleles occupy different haplotypes that are neither inherited together nor exclusive to any racial or ethnic group (Morand et al., 2023). When considering SLE phenotypes, it is important to avoid essentializing populations (Lewis et al., 2023), by conflating race with whole or proportional continental ancestry (Siddiqi et al., 2021). Since there is more intra-than inter-population genetic variation, variants associated with disease very rarely have any relationship to socially-defined races (Sirugo et al., 2021). Using proportional continental ancestry to predict disease phenotype is therefore a coarse and imprecise tool presented as a proxy for the presumed presence of one or more risk alleles. A more accurate approach would be to identify causal variants and their allelic frequencies within and between populations. The assumption that races are genetically discrete, and that genetic differences explain health disparities could lead researchers to miss factors that more substantially contribute to disparities (Pryor et al., 2021). This may also reinforce the racial stereotypes that further compound health disparities (Sankar et al., 2004).

SLE is a complex phenotype with multiple, coalescing traits and contributory immunologic underpinnings (Morand et al., 2023). Therefore, disease susceptibility and presentation are influenced both by environmental and various genetic risk factors (Vara et al., 2022). Like the disease itself, genetic risk factors for SLE are quite heterogeneous often affecting aspects of immune complex clearance, intra- or extracellular interferon (IFN) signaling pathways, and/or the adaptive immune response (Deng and Tsao, 2010). SLE is a genetically complex disease with multiple small to moderate risk loci--most of which have odds ratios between 1.1 and 1.7 (Table 1) (Niewold, 2015). Although most SLE genetic risk factors are shared, some SLE susceptibility loci may have higher or lower regional allelic frequencies (Morand et al., 2023). Therefore more inclusive SLE GWAS performed throughout the African diaspora could improve our understanding of SLE pathogenesis (Lanata et al., 2021).

**TABLE 1:** A sampling of reported SLE-associated risk alleles, their roles, and genomic locations adapted with permission (Harley et al., 2009).

SLE risk alleles	Pathway	Genomic location	Odds ratio	Ref.
PTPN22	Immune Signal Transduction	1p13	1.5	Deng and Tsao (2010)
C1q	Phagocyte/Antigen presentation	1p36.12	2.3	Wang et al. (2022)
FcGR2A	Phagocyte/Antigen presentation	1q23	1.4	Deng and Tsao (2010)
FcGR2B	Immune Signal Transduction	1q23.3	1.7	Verbeek et al. (2019)
STAT4	Type I interferon production	2q33	1.5	Deng and Tsao (2010)
STAT4	Immune Signal Transduction	2q33	1.6	Deng and Tsao (2010)
TREX1	Type I interferon production	3p21.31	1.7	Namjou et al. (2011)
BANK1	Immune Signal Transduction	4q24	1.4	Deng and Tsao (2010)
HLA-DR	Phagocyte/Antigen presentation	6p21.3	2	Deng and Tsao (2010)
C2	Phagocyte/Antigen presentation	6p21.33	1.7	Chen et al. (2015)
TNFAIP3	Type I interferon production	6q23	1.7	Deng and Tsao (2010)
IKZF1	Immune Signal Transduction	7p12.2	1.2	Deng and Tsao (2010)
IRF5	Type I interferon production	7q32	1.5	Deng and Tsao (2010)
C8orf13-BLK	Immune Signal Transduction	8p23.1	1.4	Deng and Tsao (2010)
CD274	Immune Signal Transduction	9p24.1	1.1	Chen et al. (2015)
IRF7	Type I interferon production	11p15.5	1.8	Chen et al. (2015)
ETS1	Phagocyte/Antigen presentation	11q24.3	1.3	Deng and Tsao (2010)
ITGAM	Phagocyte/Antigen presentation	16p11.2	1.4	Deng and Tsao (2010)
C3	Phagocyte/Antigen presentation	19p13.3	1.4	Miyagawa et al. (2008)
IRAK1	Type I interferon production	Xq28	1.1	Deng and Tsao (2010)

The most striking shared association across multiple rheumatic disease, including SLE, is the *HLA* region on chromosome 6, which is rich in coding genes essential to immune function (Ghodke-Puranik and Niewold, 2015). The class I and II regions encode polymorphic *HLA* genes *A, B, C*; and *DR, DQ, DP* respectively which participate in peptide antigen presentation for recognition by T-cells (Ghodke-Puranik and Niewold, 2015). Functional variation in *HLA* across ancestral populations is common owing to the variety of global infectious evolutionary pressures (Lanata et al., 2021). Although *HLA* has been most strongly associated with SLE risk in European and Chinese cohorts, it is highly polymorphic and allelic diversity is strong likely due to balancing selection—a process that actively maintains multiple alleles within a genetic pool at larger frequencies than would be expected due to genetic drift (Morris et al., 2012; Lanata et al., 2021). Fine mapping of the *HLA* region in 1,494 African American SLE cases and 5,908 controls revealed relatively short-range linkage disequilibrium (LD), with a strong association detected at the *HLA* class II region. The most significantly associated *HLA* signals differed between White and African American participants, with *HLA-DQB02:01* identified as most significant in White and *HLA-DRB115:03* in African American individuals (Hanscombe et al., 2018). In a South African SLE cohort of 45 Black patients compared to 74 ethnically matched controls, the reported prevalence of HLA-DRB1*02 is was increased in SLE cases (OR: 3.7) and was associated with serum anti-Ro antibodies (Rudwaleit et al., 1995).

High serum type I interferon (IFN) has been identified as a heritable trait that is associated with SLE risk (Niewold, 2015). Since certain subsets of SLE patients exhibit elevated serum IFN levels and more severe disease, recent research employing case-case trans-ancestral fine mapping approaches has identified new loci associated with high or low IFN phenotypes in SLE (Ghodke-Puranik et al., 2020). High IFN expression has been associated with younger age of onset, higher disease activity, SLE nephritis, and anti-RNA binding serologic markers (King A et al., 2023). Literature reports have suggested that high type I IFN is related to African ancestry, however the use of broad ancestry informative markers or self-reported and investigator-presumed racial categories is a major limitation. More recently B-cells lacking IgD and CD27, so called double negative (DN) B-cells, have been shown to be expanded particularly in African American SLE patients with high serum interferon (Jenks et al., 2018). These DN2 cells contribute to anti-Smith and RNP-producing plasma cells and are driven by extra-follicular TLR7 activation (Jenks et al., 2018). Interestingly, similar extrafollicular DN2 B-cell activation has been described during COVID-19 infection raising the possibility that infection exposure contributes to breaks in self-tolerance and SLE risk (Sachinidis and Garyfallos, 2021).

Still other ancestry-specific polymorphisms influence the likelihood of organ injury, such as lupus nephritis (LN) end stage kidney disease (ESKD). Two coding change variants in the Apolipoprotein L1 (*APOL1*) gene, G1 (*S342G* and *I384M*) and G2 (*N388del; Y389del*), were recently identified predominantly in sub-Saharan African genomes (Limou et al., 2014). Compared to the native G0 allele, the G1 and G2 variants cause cytotoxicity through disruption in mitochondrial ATP production and autophagic flux (Ashira et al., 2022). The variants are thought to have been evolutionarily conserved due to protection against *Trypanosoma brucei*, the parasite causing African trypanosomiasis (Limou et al., 2014). The *APOL1* high risk genotype (*HRG*), defined as two variants in any combination (G1/G1, G1/G2, or G2/G2), is associated with several adverse phenotypes, most notably ESKD in LN (Papeta et al., 2011; Freedman et al., 2014; Kruzel-Davila et al., 2016), where odds ratios range from 2.5 to 14, representing one of the most impactful genetic risk factors discovered (Freedman et al., 2014; Blazer et al., 2017; Blazer et al., 2021). These data highlight the importance if inclusivity in genetic research, as understanding within-ancestry genetic risks may not only impact risk stratification and personalized medicine, but also help to elucidate mechanisms of SLE disease.

**FIGURE 1:**
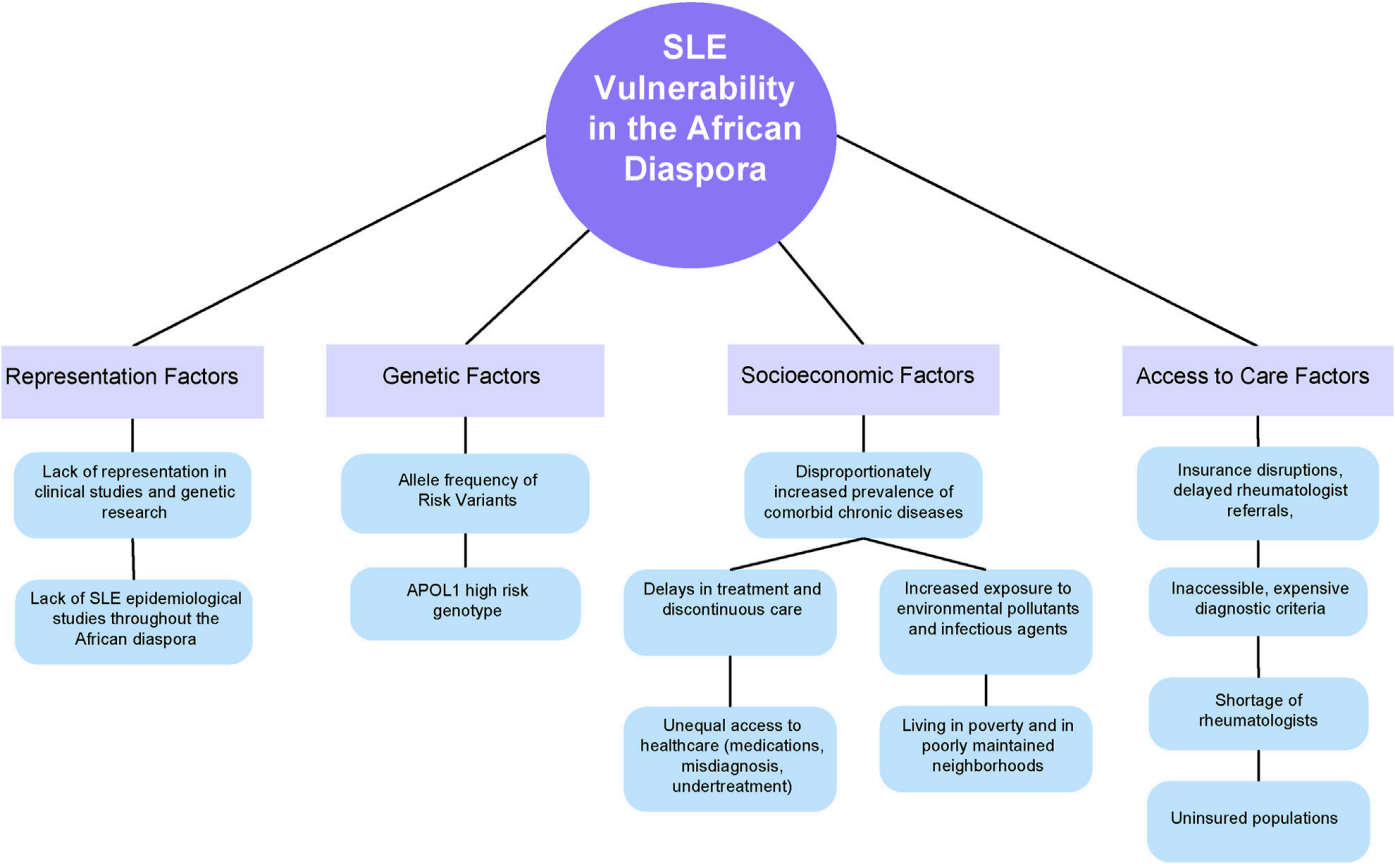
A conceptual model of the factors that lead to disproportionate SLE vulnerability throughout the African Diaspora.

Despite advancements in our understanding of the genetic basis of systemic lupus erythematosus (SLE), the inheritance patterns of the disease remain largely elusive (Morand et al., 2023). SLE is a multifaceted condition with complex inheritance mechanisms that do not adhere to Mendelian principles (Lanata et al., 2021). While over 130 genetic loci have been identified as associated with SLE, they collectively explain only a portion of the disease inheritance (Lanata et al., 2021). Given the diverse genetic architectures, gene-gene interactions, gene-environment interactions, and variability in linkage disequilibrium across populations worldwide, the predictive value of risk single nucleotide polymorphisms (SNPs) varies significantly (Lanata et al., 2021). Although disease prevalence and severity vary across populations, most Genome Wide Association Studies (GWAS) have been conducted on patients of European and Southeast Asian origin, with inadequate representation of other ancestries. Acquiring more GWAS data from African populations would be especially prudent, as the reduced linkage disequilibrium (LD) blocks compared to other global populations could make identifying causal SNPs within a candidate sequence more efficient (Park, 2019). Moreover, African genomes exhibit considerable genetic diversity, intricate genetic structures, and lower levels of linkage disequilibrium between loci compared to non-African genomes, rendering the notion of a universally shared, racially determined inherited risk improbable (Campbell and Tishkoff, 2008). Since race is not reliably measured, genetically discrete or biologically meaningful, it is more plausible that the social determinants of health explain racial disparities (Figure 1).

### SLE disease phenotypes

SLE is a heterogeneous disease which may vary from mild or moderate to extremely severe and life-threatening (Morand et al., 2023). Moreover, due to its systemic, chronic nature, SLE and its treatments are associated with comorbid chronic diseases including hypertension, diabetes, cardiovascular disease, osteoporosis, and chronic kidney disease (Barnado et al., 2018a; Sandhu et al., 2020). SLE morbidity and mortality may therefore be related to disease activity itself or the sequela of these comorbidities—commonly measured through the systemic lupus international collaborating clinics American College of Rheumatology Damage Index (SLICC/ACR DI) (Gladman et al., 1996).

Due to the living legacies of colonialism and chattel slavery, African people around the world face deep socioeconomic challenges, discriminatory practices, and structural racism (Beech et al., 2021; Goubert, 2022). Both historical and contemporary forms of colonialism result in systematic violence, wealth extraction, and profiteering which all impact the ability of indigenous populations to seek and maintain health (Tangcharoensathien et al., 2024). The social determinants of health, defined by the World Health Organization as “the conditions under which people are born, grow, work, live, and age” heavily influence SLE presentation, phenotype, and disease course (Williams et al., 2023). In the United States, minorities live in segregated neighborhoods due to redlining and discriminatory housing practices (Woo et al., 2019; Martz et al., 2021; Christensen et al., 2022; Liang et al., 2024). Neighborhood dysfunction is independently associated with self-reported disease severity and depression among Black women with SLE (Martz et al., 2021). Polluting industrial sources (air pollution, pesticides, byproducts of plastics) are also more proximally located to these neighborhoods (Gee and Payne-Sturges, 2004). Consequently, non-White communities bear a heavier burden of neighborhood dysfunction and environmental exposures which may contribute to chronic disease risk. Disadvantaged neighborhoods may also leave residents vulnerable to infectious diseases (Blazer et al., 2020; Dellaripa et al., 2024). These factors are increasingly recognized for their ability to modify SLE disease risk and pathogenesis (Huang et al., 2023; Dellaripa et al., 2024). For example, several models have linked environmental exposures to DNA damage, increased oxidative stress, and neoantigen production (Parks et al., 2017; Huang et al., 2023). These factors, and other non-genetic determinants of disease must be considered to understand SLE presentation across multiracial/ethnic groups.

#### Clinical presentation

Epidemiological studies worldwide have consistently demonstrated that SLE patients throughout the African Diaspora experience an earlier onset of the disease and more severe symptoms. In a large multi-ethnic cohort study conducted in the United States, African American ethnicity and male gender were associated with a higher lupus severity index, which measures disease severity based on a combination of established criteria (Ghalib et al., 2016). Similarly, the 1,000 Canadian Faces of SLE study revealed more severe disease manifestations in Afro-Caribbean patients, with a significant proportion experiencing neurological, hematological, and renal complications (Peschken et al., 2009). Additionally, primary Caribbean cohort studies have reported a 15% prevalence of neuropsychiatric complications, with psychosis, ischemic stroke, and seizures being the most common manifestations (Flower et al., 2017). The age of onset of SLE varies across different regions and has been reported as approximately 35–36 years in the United States, around 30 years in Canada, and between 20 and 29 years in Barbados and 29 years in Sub-Saharan Africa (Cooper et al., 2002; Peschken et al., 2009; Flower et al., 2012; Merola et al., 2014; Adelowo et al., 2021) Interestingly, in St. Lucia age of onset was reported at 26 years in impoverished 35 years in non-impoverished patients (King A et al., 2023).

Lupus nephritis (LN) has been reported as the most common and morbid SLE complication throughout the African Diaspora. In the United States, a national Medicaid beneficiary database found that the prevalence of LN was four times higher among African American women compared to White women (Feldman et al., 2013). Similarly, among a cohort of 1,537 Canadian SLE patients, Afro-Caribbean participants had more frequent renal involvement (60%) compared to White participants (40%) (Peschken et al., 2009). Primary Caribbean cohort studies in St. Lucia and Barbados have shown that 48% of participants developed nephritis within the first 3 years of diagnosis (Flower et al., 2006), whereas Sub-Saharan African cohort studies have found that 33%–50% of participants develop nephritis (Dzifa et al., 2018; Adelowo et al., 2021).

#### Comorbidities

Comorbid chronic diseases significantly contribute to poorer outcomes in African SLE patients throughout the diaspora; who exhibit elevated rates of overall organ damage accumulation compared to other racial and ethnic groups (Hasan et al., 2022). These include cardiovascular risk factors such as obesity, hypertension, and diabetes (Rhew et al., 2009; Greenstein et al., 2019; Osaze et al., 2023). African American individuals diagnosed with SLE are significantly more prone to hypertension, with a 4.25-fold higher likelihood compared to White patients. They are also at a substantially increased risk of requiring renal dialysis, with a 10.90-fold higher likelihood, and of developing pneumonia, with a 3.57-fold higher likelihood, compared to White patients (Barnado et al., 2018b). These trends have also been shown in a predominantly Black South African SLE cohort where 80% were reported to have at least one comorbidity (Greenstein et al., 2019; Osaze et al., 2023). Kidney, cardiovascular, and infectious diseases are independently associated with SLE related mortality (Rhew et al., 2009).

Unequal access to healthcare directly contributes to comorbidity risk for SLE patients throughout the African Diaspora. African Americans experience racial discrimination and low socioeconomic status which are linked with increased risk of death in SLE patients (Chandler et al., 2023; Chandler et al., 2023). Moreover, health practitioners are less likely to prescribe disease modifying anti-rheumatic drugs (DMARDS) and more likely to prescribe glucocorticoids to Black patients (Chandler et al., 2023; Williams et al., 2023). These patients are also less likely to have consistent access to rheumatologic care, and more likely to visit emergency rooms where high dose glucocorticoids are also often prescribed (Chandler et al., 2023). Chronic high-dose glucocorticoid use contributes to infection and cardiovascular disease--the leading causes of death in SLE (Barber et al., 2021). These drugs independently associate with damage accrual regardless of clinical or serological disease activity (Apostolopoulos et al., 2020). Therefore, excessive use of glucocorticoids coupled with limited access to DMARD therapy, may serve as a shared risk factor for disease severity among Black individuals with SLE across the African diaspora.

### Socioeconomic challenges

#### Poverty

Poverty significantly influences SLE incidence, prevalence, and outcomes. Among US Medicaid beneficiaries with SLE, the prevalence of LN was 16% higher in the lower socioeconomic status (SES) groups compared to the highest SES group. Moreover, living in poverty at any point was associated with greater SLE activity, accumulated damage, and poorer quality of life (Yelin et al., 2017). Impoverished participants had an average Brief Index of Lupus Damage (BILD) of 1.98 compared to 1.36 in those who had never been impoverished. Leaving poverty 2 to 3 years and 5–11 years before the baseline assessment associated with lower BILD scores at 1.44 and 1.08 respectively (Yelin et al., 2017). These trends hold throughout the African diaspora. In a cohort of 143 St. Lucian SLE patients, poverty was associated with younger age of SLE onset, higher glucocorticoid use, higher lupus severity index, and a 10% lower 3-year survival (King A et al., 2023).

The principal mechanisms by which poverty impacts SLE outcomes remain unclear. However, potential mechanisms may involve higher levels of damage accumulation resulting from glucocorticoid dependence, limited access to care, and reduced self-efficacy (Yelin et al., 2018). In the Caribbean, both poverty and educational attainment were found to exacerbate SLE outcomes. A St. Lucian study of 143 SLE patients that utilized eligibility for government assistance as a proxy for poverty found that 33% of SLE patients were unable to afford consultations, routine tests, and medications. This led to appointment cancellations and reduced adherence, defined as not taking SLE medications >80% of the time and/or by defaulting from scheduled visits more than 20% of the time. Similarly, participants with less than a tertiary education or were impoverished were less adherent at an odds ratio of 2.7 (*p* = 0.01), and 2.4 (*p* = 0.03) respectively. These patients were also more likely to have severe disease as defined by nephritis/cerebritis/dialysis at an odds ratio of 3.5 (*p* = 0.003) (Dey et al., 2021).

The cost of managing SLE is a significant barrier to care for SLE patients in Sub-Saharan Africa. This region contains many of the world’s most resource limited nations with high poverty, unemployment, and illiteracy indices (Azevedo, 2017). As there is no social security or universal, affordable health insurance in most African cities, most patients pay for their treatments out-of-pocket, often leading to delays in diagnosis and treatment and grea ter complications, morbidity, and mortality (Ranabhat et al., 2019). However, serological tests, biopsies, frequent medical visits, and medications are critical for SLE patients. The mean annual cost of managing SLE is estimated to range from $7,740.19 to $10,984, possibly increasing up to $49,754 for patients experiencing severe flares (Katz et al., 2020; Fatoye et al., 2021). These costs are a barrier in access to care, especially for patients in Sub-Saharan Africa and other resource-limited countries that lack adequate health insurance systems and have restricted access to medical services (Nicholas and Deji, 2023). Moreover, 92% of SLE patients were found to have stopped working a year after diagnosis due to their condition compared to 40% of controls (Campbell et al., 2009). SLE patients experienced greater work disability and absenteeism than healthy individuals, which impacted their prospects of being employed (Tammy O Utset et al., 2015). Therefore, the lack of affordable, accessible health insurance and employment opportunities provide significant challenges to being treated for SLE.

### Access to care challenges

SLE is a lifetime chronic illness requiring continuous access to quality primary and rheumatologic care. SLE patients throughout the African diaspora are less likely to have care continuity owing to insurance disruptions (Brown et al., 2020). After diagnosis, African Americans experience delayed rheumatology referrals compared to White and Hispanic Americans, with 64% of African Americans seeing a specialist within 3 months, in contrast to 92% of White patients and 85% of Hispanic patients. Additionally, lower education levels correlate with fewer timely rheumatology referrals, with only 45% of individuals with a high school education or less receiving timely referrals compared to 81% of those with higher education levels (Gaynon L et al., 2016). In the Caribbean, rheumatologic care faces significant cost barriers. While public care and medications are largely free in Barbados and Martinique, only 21% of Barbadians have health insurance (Flower, 2018). Conversely, St. Lucia and many smaller Caribbean islands suffer from underfunded public health systems, where essential medications listed in the public formulary are often unavailable in public pharmacies, forcing exempted patients to purchase them privately.

The cost of care poses a significant barrier for Sub-Saharan African SLE patients, especially in Nigeria where over 40% of the population lives below the global poverty level (Atobatele et al., 2022). Limited access to medications due to financial constraints, coupled with inadequate insurance coverage and state healthcare budgets, exacerbates the issue (Yenet et al., 2023). Furthermore, Sub-Saharan Africa faces challenges with inaccessible healthcare facilities, diagnostic tests, and medications, with only about 30,000 healthcare facilities serving a population of 200 million in Nigeria (Mody, 2017). Therefore, patients with rheumatologic diseases initially present at alternative health centers and are often misdiagnosed, resulting in late diagnoses, undertreatment, and development of comorbidities (Nicholas et al., 2022). Insufficient diagnostic centers and limited access to diagnostic tools, compounded by understocked pharmacies and inadequate intensive and emergency care facilities, further impede access to care in the region (Mody, 2017; Atobatele et al., 2022; Nicholas et al., 2022; Yenet et al., 2023). Disruptions in care may influence disease pathogenesis by accelerating damage accrual and disease chronicity.

### Call to action

Both a genetic propensity toward disease and social determinants influence SLE risk, presentation, and outcomes. Challenges of treating SLE across the African diaspora limit research participation in this group, which remain woefully underrepresented in GWAS studies and other clinical trials. Further as healthcare disparities overwhelmingly drive outcomes throughout the African diaspora, understanding more modest genetic influences, and gene-environment interactions becomes more challenging. The disparities in epidemiological data, access to care, diagnostic criteria, medication affordability, and workforce shortages underscore the urgent need for multifaceted interventions. We propose the following actions to advance SLE care and research within diasporic communities:

1. Invest in Epidemiological Studies: High-quality epidemiological studies are essential for comprehensively assessing the burden of lupus across the African diaspora including the United States, Caribbean, Canada, South America, and Sub-Saharan Africa. For example, the current SLE prevalence data in Sub-Saharan Africa was estimated through seven studies conducted in just six countries between 2013 and 2020. These heterogenous studies represent a narrow assessment of the 46 countries in this region, and data acquisition variation limits their comparability. Efforts should be made to expand and diversify research endeavors, particularly in underrepresented regions, to improve our understanding of SLE incidence, prevalence, and outcomes. Moreover, consistent and comprehensive collection of social determinants of health data could further our understanding of health disparities and the impact of income, discrimination, and healthcare access on SLE pathogenesis. Patient-physician partnerships through the setting up of support groups can enhance access to care and improve patient ownership of treatment decisions, ultimately leading to better adherence and long-term outcomes. Initiatives such as the Rheumatology Initiative (tRi) in Ghana demonstrate the potential for community involvement in developing units and research.
2. Enhance Diagnostic Criteria: Develop diagnostic criteria for SLE that are accessible, cost-effective, and applicable across diverse settings. By incorporating inexpensive and readily available diagnostic elements, we can facilitate early detection and expedite access to appropriate care, particularly in low- and middle-income countries (LMICs). Support groups in the Caribbean have contributed to the holistic care of SLE patients, highlighting the importance of community engagement in improving diagnostic processes.
3. Address Medication Affordability: Medication costs may be mitigated by advocating for the development and subsidization of affordable formulary medications, including disease-modifying anti-rheumatic drugs (DMARDs). Collaborative efforts with pharmaceutical procurement services and international organizations can help ensure equitable access to essential medications, particularly in resource-constrained regions. Several programs through the Caribbean Association for Rheumatology and The Rheumatology Initiative in St. Lucia and Ghana respectively, employ community cost-sharing to ensure patients maintain access to care. Through global collaboration, these initiatives could be expanded to promote health equity.
4. Expand Rheumatology Training: Scale up rheumatology training programs in LMICs to address the shortage of rheumatologists. Establishing international partnerships and leveraging technology-enabled learning platforms can enhance training opportunities and supervision, ultimately strengthening the rheumatology workforce and improving patient care. Patient involvement in clinical trials and research can also be facilitated through education and training programs for healthcare providers and community advocates.
5. Leverage Community Engagement: Engage underrepresented populations across the diaspora through community-based initiatives to increase awareness of SLE clinical and translational research and promote research participation. Collaborative endeavors, such as faith-based educational models and telemedicine services, can bridge communication gaps and facilitate wider access to research opportunities. Despite the challenges, innovative approaches to collaborative regional research, such as those championed by the Caribbean Association for Rheumatology (CAR), offer promising avenues for overcoming logistical, regulatory, and socioeconomic barriers to conducting clinical trials.

By embracing these actionable steps with commitment and collaboration, we can strive towards equitable SLE care, which is an essential bedrock for clinical and translational research. These inclusive practices can improve the quality and breadth of genetic studies throughout the African diaspora and ultimately improve the quality of life for those affected by this complex autoimmune condition.
